# The Epigenetics of Sepsis: How Gene Modulation Shapes Outcomes

**DOI:** 10.3390/biomedicines13081936

**Published:** 2025-08-08

**Authors:** Giulia Pignataro, Cristina Triunfo, Andrea Piccioni, Simona Racco, Mariella Fuorlo, Evelina Forte, Francesco Franceschi, Marcello Candelli

**Affiliations:** 1 Emergency, Anesthesiological and Reanimation Sciences Department, Fondazione Policliclinico Universitario A. Gemelli-IRCCS, 00168 Rome, Italy; andrea.piccioni@policlinicogemelli.it (A.P.); simona.racco@policlinicogemelli.it (S.R.); mariella.fuorlo@policlinicogemelli.it (M.F.); evelina.forte@policlinicogemelli.it (E.F.); francesco.franceschi@policlinicogemelli.it (F.F.); marcello.candelli@policlinicogemelli.it (M.C.); 2 Medical and Surgical Translational Medicine Department, Faculty of Medicine and Surgery, Università Cattolica del Sacro Cuore, 00168 Rome, Italy; cristina.triunfo01@unicatt.it

**Keywords:** sepsis, epigenetics, DNA methylation, histone modifications, ncRNAs

## Abstract

Sepsis is a complex and heterogeneous condition, arising from a disrupted immune response to infection that can progress to organ failure and carries a high risk of death. In recent years, growing attention has been paid to the role of epigenetic mechanisms—including DNA methylation, histone modifications, non-coding RNAs, and RNA methylation—in shaping immune activity during sepsis. These processes affect immune functions such as macrophage polarization, cytokine release, and the exhaustion of immune cells, and they help explain the shift from an initial phase of overwhelming inflammation to a later state of immune suppression. Epigenetic alterations also contribute to tissue-specific damage, notably in the lungs, kidneys, and heart, and have been linked to disease severity and clinical prognosis. Advances in transcriptomic and epigenetic profiling have made it possible to distinguish molecular subtypes of septic patients, each with distinct immune features and varied responses to treatments such as corticosteroids and metabolic therapies. Emerging biomarkers—like AQP5 methylation, histone lactylation (H3K18la), and m^6^A RNA methylation—are opening new options for patient classification and more tailored therapeutic strategies. This review examines the current understanding of how epigenetic regulation contributes to the pathophysiology of sepsis and considers its implications for developing more individualized approaches to care.

## 1. Introduction

Sepsis is a life-threatening condition marked by organ failure due to an imbalanced host reaction to infection [1], ranking among the top causes of in-hospital mortality globally. Its pathophysiology involves a complex interaction between pathogens and the host’s defenses; explaining these mechanisms is needed to progress in both knowledge and therapies, ultimately enhancing survival rates and long-term outcomes for patients. The 2023 Surviving Sepsis Campaign [2] outlined research priorities to deepen our grasp of sepsis as a multifaceted syndrome. Among these, the influence of genetic and epigenetic factors on sepsis onset, progression, and treatment response stands out as a particularly promising area. Epigenetic modifications, for instance, appear instrumental not only in modulating the immune response to infection but also in driving the immunoparalysis typical of advanced sepsis, with direct consequences for patient recovery. This review synthesizes current evidence on these mechanisms and exposes areas of uncertainty that deserve further exploration.

## 2. Search Strategy

Articles were identified using PubMed, Scopus, and EMBASE databases, through a comprehensive search conducted by combining the following key terms: ‘sepsis’, ‘epigenetics’, ‘histone modifications’, ‘DNA methylation’, and ‘non-coding RNAs’. English-language articles were screened for relevance individually by the authors, who then compared their results to include only the most relevant and recent articles. Case reports, letters, and opinions were excluded. Only articles published from 2010 onwards were included in the review.

## 3. Epigenetics

Epigenetics links environmental influences and genetic inheritance, modulating gene expression in ways that impact disease susceptibility, metabolic pathways, and stress adaptation [3]. The most studied epigenetic mechanisms operating in human cells are DNA methylation, histone post-translational modifications, and regulatory non-coding RNAs [4]. DNA methylation primarily targets cytosine residues adjacent to guanines (CpG) sites, where DNA methyltransferases (DNMTs) transfer a methyl group to cytosine’s fifth carbon, forming 5-methylcytosine (5mC). De novo methylation is catalyzed by DNMT3A and DNMT3B, whereas DNMT1 preserves methylation patterns during DNA replication by recognizing hemi-methylated strands. When CpG islands in gene promoters are methylated, transcription is typically suppressed due to the recruitment of repressive complexes by methyl-CpG binding proteins, which block the transcriptional machinery [3]. Histones form the core structure of chromatin, with nucleosomes composed of H2A, H2B, H3, and H4 subunits, and associated with linker histone H1. Covalent modifications (e.g., acetylation, methylation) of their N-terminal tails alter chromatin compaction and influence DNA accessibility for transcription, replication, and repair [4]. There are many types of histone modifications [4,5,6]. Histone methylation targets specific lysine residues (particularly on histone H3 tails) through histone methyltransferases. This modification exerts dual regulatory roles: activating transcription at H3K4, H3K26, and H3K79 sites, while silencing genes when occurring at H3K9, H3K27, or H4K20 loci. Histone acetylation, catalyzed by histone acetyltransferases (HATs), adds acetyl groups to lysines. This neutralizes their positive charge, reducing histone-DNA binding affinity and promoting transcriptional activation. Other less common modifications are the following:

- Citrullination: Mediated by peptidyl arginine deaminases (PADs), converts arginine to citrulline.
- Ubiquitination: Involves mono/poly-ubiquitin attachment to lysines, critical for genome stability.
- Lactylation: A newly characterized acylation where lactyl groups modify lysines via P300, functionally distinct from acetylation or succinylation.
- O-GlcNAcylation: A post-translational modification involving the attachment of O-linked N-acetylglucosamine moieties to serine or threonine hydroxyl groups.

This spectrum of covalent modifications regulates chromatin architecture and gene regulation through biophysical and biochemical mechanisms. The modulation of histone post-translational modifications (HPTMs) depends on three specialized enzyme classes [7]. Reader proteins identify specific histone marks, such as methylated or acetylated residues. They then recruit effector complexes to modify chromatin states. Writer enzymes catalyze the addition of new chemical groups to histone tails. In contrast, eraser proteins reverse these modifications by removing them. Examples include demethylases, which strip off methyl groups, and deacetylases, which eliminate acetyl marks. Beyond protein-coding genes, non-coding RNAs (ncRNAs) comprise a broad category of transcripts that perform essential regulatory functions despite not encoding proteins [4]. These molecules exist as either short (<200 nucleotides) or long (>200 nucleotides) forms, each participating in gene regulation through distinct mechanisms. MicroRNAs (miRNAs), typically 19–22 nucleotides in length, are transcribed by RNA polymerase II/III and precisely modulate gene expression patterns across various pathological conditions. Their small size contrasts with their extensive influence on disease-related processes. At the longer end of the spectrum, long non-coding RNAs (lncRNAs) exceed 200 nucleotides and display remarkable functional diversity. These transcripts can reshape chromatin architecture, direct histone modifications, influence alternative splicing patterns, and participate in transcriptional regulation through complex molecular interactions. Particularly noteworthy are circular RNAs (circRNAs), which form covalently closed loops through exon back-splicing. Their unique circular structure, created by 3′-5′ ligation, provides enhanced stability and specialized regulatory capabilities compared to linear transcripts [8]. Together, these ncRNA molecules significantly expand our understanding of gene regulation, operating through mechanisms that both complement and interact with conventional protein-mediated pathways.

## 4. Epigenetic Methodologies in Sepsis Research

The recent rapid advances in the field of epigenetics are due to advanced molecular techniques. One widely used approach for analyzing DNA methylation is bisulfite sequencing, in which sodium bisulfite treatment distinguishes between methylated and unmethylated cytosines. This method allows researchers to map methylation patterns across the genome, and has revealed increased methylation at immune-related genes in patients with sepsis [9]. To investigate histone modifications, researchers continue to rely on chromatin immunoprecipitation followed by sequencing (ChIP-seq). This technique uses antibodies targeting specific histone marks—like H3K27ac or H3K4me3—to isolate DNA regions associated with these modifications in immune cells [10]. More recently, the CUT&Tag method has become increasingly popular, particularly because it requires smaller sample sizes and offers greater sensitivity. This makes it especially valuable when working with clinical samples, such as monocytes from septic patients, where signs of immunosuppressive histone marks can be detected more reliably [11]. Chromatin accessibility is typically examined using ATAC-seq, a technique that tags and sequences regions of open chromatin to reveal which parts of the genome are more active or repressed. In sepsis, this has highlighted changes near genes involved in cytokine production, such as IL-1β, where regions that normally help regulate immune responses appear unusually active [12]. To further understand how DNA folds within the nucleus, researchers use Hi-C, which captures the spatial relationships between different genomic regions. In the context of sepsis, this has uncovered disruptions in the usual interactions between enhancers and promoters, offering clues as to why some immune genes are poorly regulated during infection [13]. Non-coding RNAs are also receiving growing attention in this field. Studies of circular RNAs often involve treating RNA samples with RNase R to remove linear RNAs, followed by sequencing to identify circRNAs that may influence immune signaling [14]. For microRNAs, small RNA sequencing remains a standard method to identify changes in circulating miRNA profiles during sepsis, while newer technologies such as nanopore direct RNA sequencing are beginning to reveal not only sequence information but also epitranscriptomic modifications in these small RNAs [15]. Researchers are now applying single-cell ATAC-seq to sepsis samples to explore differences in chromatin accessibility across individual immune cells, offering a more detailed view of immune heterogeneity [16]. At the same time, mass spectrometry is being used to quantify histone modifications such as H3 citrullination, which is involved in the formation of neutrophil extracellular traps (NETs), a feature of severe infection [17]. To test the functional relevance of epigenetic changes, experimental models increasingly make use of CRISPR-based epigenome editing. This involves guiding DNA-modifying enzymes to specific genomic regions using a catalytically inactive Cas9 protein fused to epigenetic effectors [18]. By targeting these enzymes to selected sites, researchers can explore how specific modifications influence gene activity in sepsis-like conditions. Together, these approaches offer a layered view of how the immune response is regulated at the epigenetic level in sepsis. From DNA methylation and histone modifications to chromatin structure and non-coding RNA expression, the emerging data are helping to build a more comprehensive picture of immune dysfunction.

## 5. The Immunological Spectrum of Sepsis

Sepsis represents a dynamic immunological process that evolves through three distinct phases [19]. The initial hyperinflammatory response, often termed a cytokine storm, features excessive release of mediators including tumor necrosis factor alfa (TNFα), interleukine (IL)-6, and IL-10. This induces a sever lymphopenia resulting from widespread apoptosis of lymphocytes in both circulation and lymphoid tissues. The final phase is represented by immunoparalysis, which is characterized by a markedly reduced immune responsiveness to stimuli and a global impairment of host defense mechanisms. It involves a combination of decreased antigen presentation, reduced pro-inflammatory cytokine release, impaired phagocytic capacity, and profound lymphocyte dysfunction. Clinically, it is often identified by low expression of HLA-DR on monocytes and reduced ex vivo responsiveness to endotoxin, particularly a diminished TNF-α response. This immunosuppressive state increases susceptibility to secondary infections, delays wound healing, and is associated with poor long-term outcomes in septic patients. Although originally considered a passive consequence of immune exhaustion, immunoparalysis is now recognized as an actively regulated process involving multiple immune pathways and mediators.

**Figure 1:**
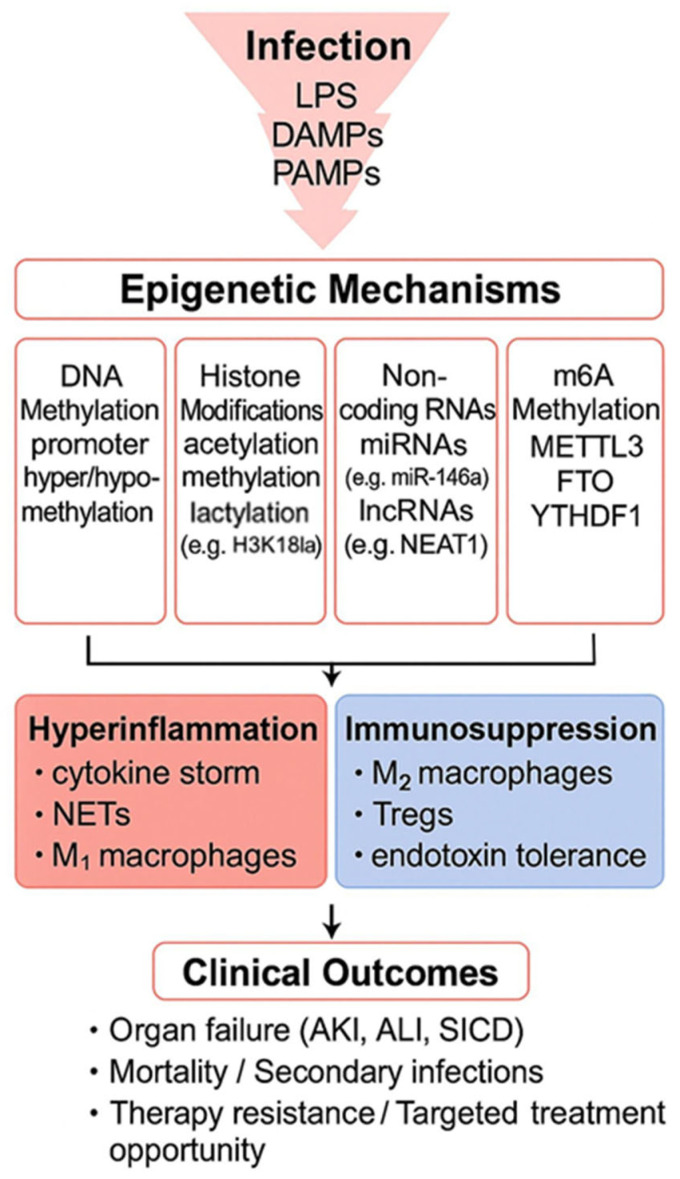
Epigenetic Modifications in Sepsis-Induced Immunosuppression. Legend: LPS: lipopolysaccharide, DAMPs: damage-associated molecular patterns, PAMPS: pathogen-associated molecular patterns, miRNAs: microRNAs, lncRNAs: long non-coding RNAs, NEAT1: Nuclear Paraspeckle Assembly Transcript, m^6^A: N6-methyladenosine, METTL3: methyltransferase-like 3, FTO: fat mass and obesity-associated protein, YTHDF1: YTH N6-Methyladenosine RNA Binding Protein 1, NETs: neutrophil extracellular traps, AKI: acute kidney injury, ALI: acute lung injury, SICD: sepsis-induced cardiac dysfunction.

While this progression reflects a homeostatic attempt to counterbalance inflammation (the compensatory anti-inflammatory response syndrome, CARS), some survivors develop persistent inflammation, immunosuppression and catabolism syndrome (PICS) [20,21]. PICS presents particular clinical challenges, including muscle wasting, delayed wound healing, heightened infection susceptibility, and increased mortality risk—all contributing to prolonged functional impairment. The underlying immunosuppression affects both innate and adaptive immunity through multiple mechanisms such as elevated anti-inflammatory cytokine production, enhanced immune cell apoptosis, autophagy activation, T-cell exhaustion, and expansion of regulatory populations like regulatory T cells (Tregs) and myeloid-derived suppressor cells (MDSCs). Tregs, a specialized CD4+ T cell subset, maintain immune equilibrium under physiological conditions. However, their expansion during sepsis—particularly when exceeding 15% of circulating CD4+ T cells—initiates detrimental immunosuppression. Clinical studies demonstrate that sepsis selectively expands forkhead box P3 (Foxp3+) Treg populations while augmenting their inhibitory capacity, with these changes directly correlating with elevated mortality rates [22]. The immunosuppressive effects manifest through several interconnected pathways. First, they involve the secretion of anti-inflammatory cytokines, such as transforming growth factor-β (TGF-β) and IL-10. Second, they induce inhibitory surface receptors on effector lymphocytes, including T-cell immunoglobulin and mucin-domain containing-3 (TIM-3), programmed cell death protein 1 (PD-1), T cell immunoreceptor with Ig and ITIM domains (TIGIT), and cytotoxic T-lymphocyte antigen 4 (CTLA-4). Third, they promote epigenetic modifications that stabilize *Foxp3* expression. Finally, they induce a metabolic shift toward oxidative phosphorylation, reducing glycolytic activity [23]. The pathophysiology of sepsis-induced immune paralysis further involves the expansion of MDSCs, comprising two distinct subsets—polymorphonuclear and monocytic variants. These immature myeloid populations exert broad immunosuppressive effects. One mechanism is nitric oxide production, which disrupts antigen presentation. This occurs through the downregulation of major histocompatibility complex (MHC) class II molecules. Nitric oxide also interferes with JAK3/STAT5 signaling pathways (janus kinase 3/signal transducer and activator of transcription 5). Additional mechanisms include generation of reactive oxygen species, induction of apoptotic pathways in lymphocytes, and depletion of the essential amino acid L-arginine from the microenvironment. Multiple clinical investigations have established that elevated MDSC levels predict poorer outcomes in sepsis patients. Particularly strong associations have been observed in cases progressing to persistent inflammation, immunosuppression, and catabolism syndrome [24,25]. A particularly significant manifestation of sepsis-induced immune dysfunction involves endotoxin tolerance. It is characterized by diminished responsiveness to repeated bacterial component exposure, particularly lipopolysaccharide (LPS) [22]. This adaptive response leads to an impaired TNF production while promoting expression of non-tolerizable genes. The resulting cytokine profile shifts from pro-inflammatory mediators (TNF-α, IL-1β) toward anti-inflammatory signals (IL-10, TGF-β), reflecting an important cellular reprogramming. The molecular basis of this phenomenon involves both cell-intrinsic and epigenetic modifications. Specific mechanisms including G protein-coupled receptor 84 (GPR84) mediated suppression of TNF mRNA in tolerant monocytes and impaired nuclear factor kappa-light-chain-enhancer of activated B cells (NF-κB) phosphorylation capacity has been described in septic patients [26]. These alterations collectively contribute to the immune paralysis observed in advanced sepsis. Initially viewed as a protective, anti-inflammatory adaptation to prevent excessive tissue damage, endotoxin tolerance is now understood as a complex reprogramming of the immune response that may contribute to the immunosuppression seen in sepsis. It represents a selective, not global, downregulation of inflammatory signaling, and plays a critical role in balancing host defense with the risk of immunopathology during systemic infection. Emerging research highlights how innate immunity can develop memory-like responses through epigenetic reprogramming of transcriptional pathways. This process, also known as trained immunity, occurs in response to many endogenous and exogenous stimuli [4]. The discovery of such mechanisms has changed our understanding of immune adaptation during critical illness. Current evidence increasingly supports the importance of epigenetic modifications in inducing sepsis-related immunosuppression as well as initial hyperinflammation, as shown in Figure 1. These molecular changes influence disease progression, therapeutic responses, and ultimately clinical outcomes.

## 6. DNA Methylation

The regulation of gene expression through DNA methylation serves to control immune responses during sepsis. Altered methylation patterns contribute to both the hyperinflammatory and immunosuppressive phases of the disease. Hypomethylation of specific genes can potentiate the cytokine storm characteristic of early sepsis, whereas hypermethylation of cytokine-related genes may induce subsequent immune suppression [27]. This epigenetic reprogramming results in selective modulation of immune pathways—for instance, upregulating IFN-γ signaling while silencing other protective responses [22]. These methylation changes exert clinically significant effects. Methylation of genes regulating nitric oxide production contributes to the endothelial dysfunction observed in sepsis, promoting pathological vasoconstriction and tissue ischemia [27]. Similarly, hypermethylation of the *aquaporin-5* (AQP5) *promoter* reduces expression of aquaporin 5, eliminating its protective effects in septic patients [28]. The plasticity of DNA methylation patterns allows responses to diverse stimuli. Endogenous factors like cytokines and exogenous triggers including microbial components can modulate the methylome. Pathogens such as *H. pylori* have this capacity through induction of hypermethylation in gastric mucosal genes, including tumor suppressors and DNA repair genes, thereby promoting carcinogenesis [29]. Viral pathogens including H5N1 influenza and Middle East respiratory syndrome Coronavirus (MERS-CoV) similarly subvert host defenses by methylating promoters of antigen presentation genes, effectively dampening immune recognition [30]. This epigenetic regulation represents a double-edged sword in sepsis—while potentially adaptive in moderating inflammation, it may also perpetuate immunosuppression and organ dysfunction. The pathogen-specific modifications further illustrate how microbial evolution has co-opted host epigenetic machinery. A study by Beltrán-García et al. [31] provides important insights into the dynamic DNA methylation changes occurring during sepsis progression. The authors performed a comparative analysis of leukocyte methylation patterns across three groups: septic patients, septic shock patients, and critically ill controls. They identified significant alterations in Differentially Methylated Positions (DMPs). These methylation changes specifically impacted several immune regulators, including IL-10, triggering receptor expressed on myeloid cells 1 (TREM1), IL-1B, and TNFα-Induced Protein 8 (TNFAIP8). Notably, patients with septic shock exhibited distinct hypomethylation patterns at promoter regions of IL-1B and TNFα compared to those with uncomplicated sepsis, consistent with enhanced pro-inflammatory cytokine production. Paradoxically, these same patients showed hypomethylation of the *IL-10* gene, suggesting concurrent activation of anti-inflammatory pathways (TGF-β, IL-10, IL-13) as part of the CARS that follows systemic inflammatory response syndrome (SIRS). These findings align with previous reports demonstrating how sepsis-induced cytokine storm affects epigenetic remodeling at pro-inflammatory gene promoters in monocytes and macrophages [26]. However, while other researchers [4] emphasize the link between DNA methylation changes and immunosuppression—proposing that DNA methyltransferase inhibitors (DNMTis) could reverse damaging epigenetic marks and enhance outcomes in animal models—this study reveals a different perspective. The findings demonstrate widespread methylation alterations in immune-related genes (including *IL-10* and *TREM1*) in septic shock patients. Yet, the research leaves unanswered whether these modifications serve an adaptive purpose, contribute to pathology, or simply reflect disease severity.

**Figure 2:**
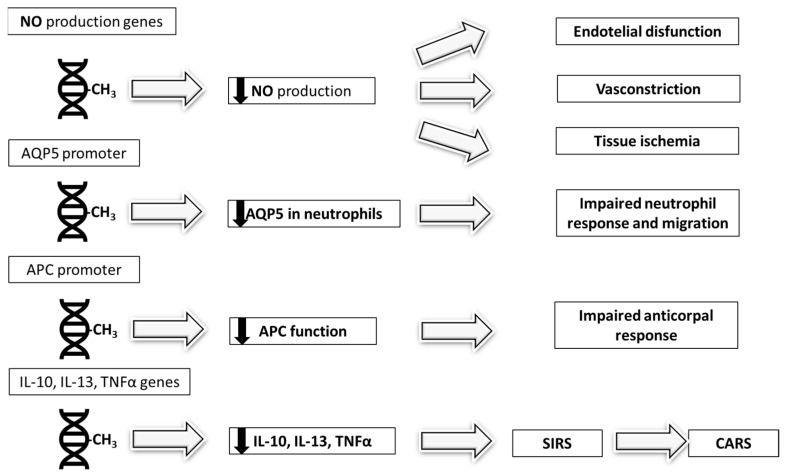
DNA methylation mechanisms involved in altered inflammatory response in sepsis. NO: nitric oxyde, -CH_3_: methy group, AQP5: aquaporin-5, APC: antigen-presenting cell, IL: interleukin, TNF: tumor necrosis factor, SIRS: systemic inflammatory response syndrome, CARS: compensatory anti-inflammatory response syndrome.

In any case, the observed methylation changes appear clinically relevant, showing correlation with established markers of disease severity, including the sequential organ failure assessment (SOFA) scores and lactate levels. This epigenetic dysregulation may contribute to the exaggerated initial inflammatory response and subsequent immunosuppression characteristic of septic shock. The dynamic nature of these methylation alterations suggests they could be used as biomarkers for disease progression and potential therapeutic targets. Recent investigations have highlighted the potential involvement of exosomes (0.05–1 μm membrane-bound vesicles) in sepsis pathophysiology. These microvesicles, released by cells in response to various stimuli, including cytokines, act as intercellular messengers by transferring bioactive molecules—in particular mRNA—capable of modifying recipient cell behavior [32]. Wisler et al. [33] provided evidence that circulating exosomes participate in immune regulation during sepsis. Their work demonstrated that these vesicles suppress monocyte TNF-α production following LPS stimulation, indicating modulation of the toll-like receptor 4 (TLR4) signaling pathways. Subsequent studies revealed broader immunosuppressive effects, including STAT3 activation and NF-κB pathway inhibition. A particularly significant finding involves the exosomal transfer of DNMT mRNA during sepsis. Research indicates these vesicles carry elevated levels of DNMT transcripts that, upon delivery to monocytes, induce TNF-α promoter methylation and subsequent gene silencing [34,35]. Dakhlallah et al. [36] further established that septic patients exhibit higher levels of DNMT1 and DNMT3A proteins within circulating microvesicles compared to non-septic critically ill controls. These epigenetic modifications contribute to monocyte dysfunction and sepsis-induced immunoparalysis. The altered methylation patterns appear to influence macrophage polarization, with DNMT3b overexpression favoring the pro-inflammatory M1-like phenotype [37]. This dysregulation of macrophage differentiation represents one of several mechanisms through which exosome-mediated epigenetic changes may perpetuate the immunosuppressive phase of sepsis. In Figure 2 are described the epigenetic mechanisms regulated by DNA methylation during sepsis.

## 7. Histone Modifications

The epigenetic profile of sepsis involves significant histone modifications that affect both innate and adaptive immune responses. These post-translational changes contribute to the profound immune remodeling observed during sepsis progression. Within the innate immune system, neutrophils and antigen-presenting cells (monocytes, macrophages, and dendritic cells) undergo specific histone-mediated changes that influence their function. Neutrophils, mediators of early hyperinflammation, deploy multiple antimicrobial mechanisms, including protease release, reactive oxygen species generation, and formation of neutrophil extracellular traps (NETs). These web-like DNA/histone structures are unique to neutrophil biology and require histone citrullination. It is a process catalyzed by peptidylarginine deaminases (PADs), with PAD4 playing a particularly important role. The activation of PAD4 itself depends on prior histone H3 deacetylation mediated by histone deacetylase 1 (HDAC1) [6,38]. Concurrently, specific histone modifications influence neutrophil survival. Elevated acetylation at the H4K16 position correlates with increased apoptotic rates in these cells [39], suggesting an additional layer of epigenetic control over neutrophil-mediated inflammation. These coordinated modifications demonstrate how histone alterations can simultaneously regulate both antimicrobial functions and cellular lifespan during sepsis.

The cumulative effect of these changes contributes to the dynamic transition from initial hyperinflammation to subsequent immunosuppression that characterizes sepsis pathophysiology. The functional impairment of monocytes, macrophages, and dendritic cells during and after sepsis involves significant alterations in histone modification patterns. These epigenetic changes contribute to both acute immune dysregulation and persistent cellular defects observed in septic patients. Distinct histone marks correlate with specific functional states in these cells. Elevated H3K4 acetylation promotes transcription of non-tolerized genes, driving a pro-inflammatory phenotype [22]. Conversely, diminished H3 and H4 acetylation coupled with increased H3K27 trimethylation suppresses macrophage effector functions [38]. The inflammatory response to LPS further modulates these patterns through activation of jumonji domain-containing protein 3 (JMJD3), a histone demethylase that removes repressive H3K27me2/3 marks, thereby enhancing expression of proinflammatory mediators like IL-1β [22]; the exposure of macrophages to the LPS has been shown to cause the translocation of the cytosolic enzyme ATP citrate lyase (ACLY), with subsequent histone modification and activation of the NF-κB pathway [40]. However, some sepsis-induced epigenetic modifications persist beyond the acute phase, contributing to long-term immune alterations. Research by Davis et al. [41] demonstrates that sustained downregulation of the histone methyltransferase mixed-lineage leukemia 1 (MLL1) leads to reduced H3K4me3 at NFκB-binding sites, resulting in prolonged suppression of inflammatory gene expression. This mechanism may underlie the persistent immunoparalysis observed in sepsis survivors. The cumulative effect of these modifications creates a complex epigenetic profile that induces both the hyperinflammatory and the immunosuppression phases influencing clinical outcomes. The impact of sepsis exerts dramatic epigenetic alterations across immune cell populations, determining both acute inflammatory responses and long-term immunological consequences. In dendritic cells, studies on post-septic murine models reveal how modified histone methylation patterns at the *IL-12* promoter—characterized by diminished H3K4 methylation and elevated H3K27 methylation—directly impair IL-12 production and suppress proinflammatory signaling [26]. Nonetheless, histone modifications also reveal inconsistencies: while H3K4me3 is considered an activating mark, its functional outcomes vary with timing and cell type, and H3K27me3 may either suppress inflammation or impair immune recovery. Experimental therapies targeting histone acetylation or methylation yield variable results across studies, with outcomes dependent on dose, disease stage, and timing.

These changes illustrate the molecular basis for dendritic cell dysfunction observed in sepsis survivors. Circulating damage-associated molecules like heme further contribute to epigenetic reprogramming during sepsis. Experimental evidence demonstrates that heme induces H3K27 acetylation at the *IL-8* promoter, inducing enhanced IL-8 expression [42]. This mechanism links tissue injury to sustained inflammatory signaling through chromatin remodeling. The adaptive immune compartment shows parallel epigenetic disturbances. T cell populations exhibit increased repressive H3K27 methylation of two transcriptional regulators—the interferon (IFN) γ locus in Th1 cells and GATA3 (guanine–adenine–thymine–adenine binding protein 3) in Th2 cells. The consequence is significantly impaired effector function in these cell populations [43]. Simultaneously, permissive histone acetylation at H3K9 within the Foxp3 locus promotes Treg differentiation and amplifies immunosuppressive activity [26,38]. These coordinated modifications create an immunological imbalance that persists beyond the acute phase of sepsis. The convergence of these epigenetic changes across innate and adaptive immunity underscores their clinical significance. From suppressed dendritic cell activation to skewed T cell differentiation, chromatin modifications emerge as central mediators of sepsis-induced immunopathology. Various pathogens can directly manipulate the function of immune cells through specific virulence factors influencing histone modifications. *L. monocytogenes* employs its listeriolysin O (LLO) toxin to induce histone H4 deacetylation and H3S10 dephosphorylation at immune gene promoters, effectively silencing host defense mechanisms. Similar histone modifications are triggered by *C. perfringens* perfringolysin and *S. pneumoniae* pneumolysin. Other pathogens have developed distinct epigenetic subversion strategies: *C. trachomatis* utilizes its nuclear ubiquitin E3 ligase (NUE) protein to methylate host histones, while *S. flexneri* deploys effector proteins like outer surface protein F (OspF) and invasion plasmid antigen H 9.8 (IpaH9.8) to interfere with histone phosphorylation and block immune gene activation [29,30]. The discovery of histone lactylation has revolutionized our understanding of lactate’s role in sepsis pathophysiology. While traditionally considered just a metabolic byproduct, lactate accumulation reflects the metabolic transition from oxidative phosphorylation to glycolysis during sepsis. This shift represents an adaptation designed to quickly satisfy immune cells’ increased energy requirements [44]. While lactate levels remain clinically valuable as biomarkers of tissue hypoperfusion and predictors of mortality [45], their biological significance extends far beyond this diagnostic role. The emerging paradigm recognizes lactate as a signaling molecule capable of modifying histones through lactylation. This post-translational modification creates a direct link between cellular metabolism and gene regulation during sepsis. The implications are intricate: as lactate levels rise, histone lactylation may serve as a metabolic sensor, translating the cellular energy state into epigenetic reprogramming of immune responses. This mechanism potentially contributes to both the early and late phases of sepsis. However, more recent studies have underlined an even more important role for lactate, particularly in contributing to the development of immunosuppression in sepsis. In 2019, Zhang et al. [46] identified the lysine lactylation (Kla), a never-before-discovered post-translational modification on histone proteins. In their experimental model, they demonstrated that lactate serves as a direct precursor for histone lactylation and that glucose levels elevate both intracellular lactate and histone Kla in a dose-dependent manner. Moreover, they found that histone Kla levels increased during the late phase of M1 macrophage polarization, suggesting that “histone lactylation acts as a “lactate clock,” modulating gene expression to promote resolution and repair following inflammation”. Among various lactylation targets (both histone and non-histone proteins), H3K18la has emerged as particularly significant in sepsis pathophysiology. Chu et al. [47] first demonstrated how H3K18la levels correlate strongly with multiple clinical severity markers in septic shock patients—including serum lactate, procalcitonin, acute physiology, and chronic health evaluation (APACHE) II and SOFA scores, along with intensive care unit (ICU) stay duration and mechanical ventilation requirements. Their findings suggest this modification may serve as both a severity marker and potential contributor to sepsis-associated immunoparalysis, given its association with elevated levels of both pro-inflammatory (IL-6) and immunosuppressive (IL-10) cytokines. Beyond its clinical correlations, H3K18la appears functionally important in macrophage polarization and occurs in M1 macrophages under hypoxic conditions or following stimulation by LPS and IFN-γ [36]. H3K18la marks genes involved in tissue repair pathways and associates preferentially with the M2-like macrophage phenotype. This pattern suggests a potential role in mediating the shift from inflammatory to reparative immune states during sepsis recovery. These findings collectively position histone lactylation, particularly at H3K18, as a molecular bridge between metabolic changes and immune dysfunction in sepsis. The modification directly couples elevated lactate levels to both the acute inflammatory phase and subsequent immunosuppression through epigenetic mechanisms. H3K18la has also been linked to sepsis-associated acute kidney and lung injuries (AKI and ALI). Qiao et al. [48] discovered an increase in Pan Kla, H3K9la, and H3K18la in total kidney proteins in CLP (cecal ligation and puncture) model mice versus controls; similarly, they also found an upregulation of global lactylation and H3K18la associated with increasing LPS concentration. Furthermore, this study revealed significant H3K18la signal enrichment at the Ras homolog gene family A (RhoA) promoter. This epigenetic modification has been linked to activation of Rho-associated coiled-coil-containing protein kinase (ROCK).

ROCK activation triggers ezrin recruitment, which can stimulate downstream pathways and induce both NF-kB upregulation and inflammatory cytokine release. The cascade culminates in kidney cellular injury. Another study indicates that lactylation at lysine 20 of *Fis1* may be the mechanism through which lactate can cause CLP-induced AKI [49]. The most important histone modifications and their effect on the pathophysiology of sepsis are included in Table 1.

Recent research has identified H3K27me3 as another important epigenetic marker in sepsis-associated AKI. This modification involves trimethylation of lysine 27 on histone H3, mediated by the histone methyltransferase EZH2 (enhancer of zeste 2 polycomb repressive complex 2 subunit). Li B. et al. [50] demonstrated that reducing EZH2 activity decreases H3K27me3 levels at the *Sox9* (SRY-Box Transcription Factor 9) gene. This epigenetic change relieves repression of *Sox9* expression, subsequently activating the Wnt/β-catenin signaling pathway. In LPS-treated mice, this cascade produced protective effects in kidney tissue by suppressing apoptotic processes and reducing inflammatory responses. The direct correlation found between H3K18la increase during sepsis-associated ALI and accelerated degradation of the glycocalyx on the surface of pulmonary vessels increases vascular permeability and exacerbates lung injury [51]. In particular, H3K18la upregulates the transcription of early growth response 1 (EGR1), increasing the production of heparanase (HPSE), an enzyme responsible for glycocalyx degradation and endothelial barrier dysfunction; interestingly, H3K18la levels correlate with sepsis-associated ARDS (Acute Respiratory Distress Syndrome) severity. The role of histone modifications in sepsis pathogenesis suggests that the enzymes mediating these changes may themselves represent promising therapeutic targets. Among these, the sirtuin (SIRT) family—comprising seven nicotinamide adenine dinucleotide+ (NAD)-dependent proteins—has attracted particular interest due to its deacetylase activity and regulatory functions. While sirtuins also participate in oxidative stress responses, a discussion of this aspect falls outside the scope of our current focus. The immunopathological transition in sepsis involves SIRT1 activation by accumulating nuclear NAD+. The predominant nuclear sirtuin, SIRT1 coordinates two complementary anti-inflammatory pathways. First, it induces NF-κB p65 deacetylation that dampens expression of cytokines like TNF-α and IL-1β [52]. Second, it drives RelB-dependent chromatin remodeling that converts active euchromatin to transcriptionally silent heterochromatin at inflammatory gene loci [53]. This chromatin reprogramming requires sequential action of SIRT1, the methyltransferase G9a (mediating H3K9me2), and DNA methyltransferases DNMT3a/3b, ultimately forming stable repressive chromatin structures [54]. Qin K et al. [55] have shown that an increase in SIRT2/SIRT5 expression ratio in macrophages can lead to a downregulation of NF-kB activation and consequently contribute to the development of endotoxin tolerance. Finally, nuclear SIRT6 and SIRT7 have been associated with H3K9 deacetylation, which represses the transcription of glycolytic genes and can modulate inflammation, and ribosomal RNA transcription and stress response during sepsis [56].

## 8. Non-Coding RNAs

In recent years, non-coding RNAs (ncRNAs)—such as microRNAs (miRNAs), long non-coding RNAs (lncRNAs), circular RNAs (circRNAs), and epitranscriptomic regulators like N6-methyladenosine (m^6^A)—have become increasingly important in understanding sepsis development. These ncRNAs regulate immune signaling, cell death, oxidative stress, and organ dysfunction by controlling gene expression at both the transcriptional and post-transcriptional levels. They also show stable expression in blood and tissues, making them potential biomarkers and targets for treatment. Beyond ncRNAs, m^6^A methylation—a common RNA modification—has been found to influence immune responses in sepsis, affecting how RNA behaves and how cells cope with stress. MiRNAs are small, naturally occurring RNAs (about 22 nucleotides long) that regulate gene expression after transcription by binding to target mRNAs, blocking their translation or marking them for breakdown. During sepsis, miRNA levels change dynamically, closely tied to inflammation and immune system activity. MiR-146a is among the most extensively investigated miRNAs in sepsis. It modulates inflammatory responses by targeting central components of the TLR/NF-κB pathway, including IL-1 receptor-associated kinase 1 (IRAK1) and TNF receptor-associated factor 6 (TRAF6). Experimental evidence indicates that elevated miR-146a expression mitigates cardiac dysfunction and suppresses pro-inflammatory cytokine production in septic models, supporting its role as a protective regulator [8]. Similarly, miR-223 influences neutrophil activity and NLRP3 (NOD-, LRR-, and Pyrin domain-containing protein 3) inflammasome function, with its expression levels associated with M2 macrophage polarization and enhanced survival in murine sepsis [57]. Conversely, miR-155 and miR-125b are upregulated during the early phases of sepsis, where they exacerbate inflammatory signaling. MiR-155 inhibits the suppressor of cytokine signaling 1 (SOCS1), leading to sustained NF-κB activation and increased cytokine release [48]. MiR-125b promotes M1 macrophage polarization by inhibiting B-cell lymphoma 6 (Bcl6), thereby amplifying c-Jun N-terminal kinase (JNK) pathway activity [37]. Zhang et al. [8] identified miR-25 and miR-150 as potential diagnostic biomarkers for sepsis. Notably, miR-25 exhibited greater predictive accuracy than conventional markers, such as C-reactive protein (CRP) and procalcitonin [21]. The same study emphasized the organ-specific regulatory roles of miR-122 and miR-142 in hepatic and renal injury, respectively, further underscoring the involvement of miRNAs in sepsis pathogenesis. LncRNAs are RNA transcripts exceeding 200 nucleotides that lack protein-coding capacity but induce chromatin remodeling, transcriptional regulation, and post-transcriptional processes. In sepsis, lncRNAs have been identified as important modulators of inflammatory responses and immune tolerance, with systemic effects across various organ systems. The nuclear-enriched abundant transcript 1 (NEAT1) is one of the most upregulated lncRNAs in experimental sepsis. It enhances inflammatory signaling by acting as a molecular sponge for miR-125a-5p, which relieves suppression of *TRAF6* and amplifies TLR4/NF-κB pathway activation. Experimental knockdown of *NEAT1* in macrophages reduces LPS-related cytokine release and promotes M2 macrophage polarization [37]. Another pro-inflammatory lncRNA, MALAT1 (metastasis-associated lung adenocarcinoma transcript 1), contributes to endothelial dysfunction in sepsis. By sequestering miR-150-5p, MALAT1 upregulates intercellular adhesion molecule 1 (ICAM-1) expression, worsening pulmonary inflammation in sepsis-associated ALI [58]. Conversely, certain lncRNAs exhibit protective effects. TUG1 (taurine-upregulated gene 1) suppresses inflammation through the miR-9-5p/SIRT1 axis, facilitates M2 macrophage polarization, and inhibits NF-κB activation [37,58]. Extracellular vesicles carrying TUG1, derived from endothelial progenitor cells, have been shown to enhance survival in septic mice by reprogramming macrophage function [37]. Li et al. comprehensively analyzed the involvement of lncRNAs in septic cardiomyopathy, identifying distinct pathological roles for myocardial infarction-associated transcript (MIAT) and HOX transcript antisense intergenic RNA (HOTAIR). MIAT induces cardiomyocyte apoptosis through the miR-330-5p/NF-κB signaling axis, while HOTAIR exacerbates cardiac inflammatory responses via MAPK pathway activation. In contrast, TUG1 has cardioprotective properties by preserving mitochondrial membrane integrity and promoting autophagic flux through SIRT1/PGC-1α-mediated mechanisms [59]. These findings collectively highlight the functional diversity of lncRNAs in sepsis pathogenesis, with both pro-inflammatory and cytoprotective actions that exhibit strict cell-type and organ specificity. The opposing effects of different lncRNAs underscore their complex regulatory networks in sepsis-associated organ dysfunction. CircRNAs constitute a unique class of endogenous RNA molecules characterized by their covalently closed circular structure formed through back-splicing. This distinctive configuration confers remarkable stability against exonuclease-mediated degradation. While their investigation in sepsis lacks that of other non-coding RNAs, increasing evidence positions circRNAs as significant modulators of the inflammatory cascade during septic responses. Beltrán-García et al. identified several circRNAs that function as competitive endogenous RNAs (ceRNAs), sequestering miRNAs to regulate inflammatory pathways. A prime example is circ_0003159, which, through its interaction with the miR-223 axis, exerts control over macrophage polarization and the production of inflammatory mediators. Parallel mechanisms involve circ_102685 and circular remodeling and spacing factor 1 (circ-RSF1), both of which engage miR-146a, indicating a potential role for circRNAs in mediating innate immune memory and endotoxin tolerance [60]. The functional spectrum of circRNAs in sepsis includes both pro-inflammatory and anti-inflammatory effects. For example, circPPM1F (circular RNA from gene protein phosphatase, Mg^2+^/Mn^2+^-dependent 1F) induces M1 macrophage polarization and amplifies NF-κB signaling. On the other hand, circ_0038644 demonstrates protective effects through suppression of IL-6 synthesis [61]. These opposing regulatory functions likely contribute to the modulation of cytokine storm intensity during severe sepsis. Current understanding of circRNA involvement in sepsis-associated organ dysfunction remains incomplete. However, preliminary findings suggest their participation in regulating immune cell trafficking and tissue injury processes. The combination of their stability and abundant expression in immune cells and circulation makes circRNAs particularly suitable for development as diagnostic biomarkers and potential therapeutic targets in sepsis management. Exosomes carry bioactive cargoes including ncRNAs that mediate intercellular signaling. Hashemian et al. reviewed the role of exosomal ncRNAs in the pathogenesis of sepsis, highlighting their involvement in macrophage polarization, endothelial dysfunction, and immune cell recruitment [57]. PMN-derived exosomes enriched in miR-30d-5p were shown to promote M1 polarization and pyroptosis via NF-κB activation, exacerbating lung injury in sepsis models. Likewise, endothelial progenitor cells (EPC)-derived exosomes containing TUG1 improved lung repair and enhanced M2 macrophage activity [58]. Because exosomal ncRNAs are present in plasma and reflect the molecular state of parent cells, they have potential as both biomarkers and therapeutic tools. Their role as vehicles of functional RNA transfer in sepsis remains a promising field for future translational research. Beyond the regulation exerted by ncRNAs, RNA metabolism is subject to epitranscriptomic modification, with m^6^A being the most prevalent internal modification of eukaryotic mRNA. The m^6^A mark influences RNA splicing, stability, translation, and localization, and it is dynamically regulated by “writers” (e.g., METTL3, METTL14), “erasers” (e.g., FTO, AlkB homolog 5, RNA demethylase -ALKBH5-), and “readers” (e.g., YTHDF) proteins. Growing evidence links m^6^A methylation to immune homeostasis and inflammatory responses during sepsis. Bi et al. proposed that dysregulation of m^6^A contributes to immune dysfunction and cytokine overproduction [62]. Specifically, METTL3 has been shown to methylate STAT1 mRNA, enhancing its translation and promoting M1 macrophage polarization [58,63]. This epigenetic shift favors the production of TNF-α, IL-6, and other inflammatory mediators, aggravating the cytokine storm. In contrast, the demethylase FTO suppresses the inflammatory response by destabilizing mRNAs involved in inflammasome activation. ALKBH5, another eraser, facilitates neutrophil migration and supports effective bacterial clearance [64,65]. These opposing roles suggest that m^6^A is a finely tuned system controlling both pro- and anti-inflammatory pathways. Chen et al. emphasized that m^6^A methylation levels are altered in septic patients and influence the transcriptome of immune cells, including monocytes and macrophages [66]. Their findings showed that altered expression of METTL3, ALKBH5, and FTO stratifies patients into different immune response phenotypes, with potential prognostic value. Wang et al. proposed that modulating m^6^A levels may offer a novel therapeutic avenue, particularly through targeted inhibition of METTL3 to reduce systemic inflammation [64]. In cardiac tissue, for example, METTL3 promotes calcium overload and mitochondrial damage during septic cardiomyopathy, while FTO activity appears protective [67]. M^6^A methylation represents a critical and dynamic control point in the immune response to sepsis. Its intersection with ncRNAs (e.g., NEAT1, which is stabilized via m^6^A modification) adds complexity to transcriptomic regulation in inflammation and organ failure. NcRNAs have been shown to also play a role in the polarization of macrophages into classically activated M1 or alternatively activated M2 phenotypes, which is a central element in the immunopathogenesis of sepsis. Several ncRNAs that drive macrophage polarization in sepsis have been described. For instance, miR-9, miR-27b, and miR-155 enhance M1 differentiation by targeting anti-inflammatory signaling molecules. In contrast, miR-146a, miR-124, miR-223, and miR-132 support M2 polarization and contribute to resolution of inflammation [37]. Essandoh et al. further emphasized the dual role of miR-21, which promotes M1 polarization early in sepsis but supports M2 transition during the resolution phase [21]. This time-dependent function illustrates the complexity of miRNA-mediated regulation. Wang et al. discussed exosomal miRNAs from neutrophils and endothelial progenitor cells that modulate macrophage fate. MiR-30d-5p in PMN-derived exosomes induces M1 polarization and pyroptosis, while TUG1-carrying vesicles from EPCs promote M2 activity via the miR-9-5p/SIRT1 axis [58]. Beltrán-García et al. highlighted that circPPM1F enhances M1 polarization through NF-κB activation, and circ_0038644 inhibits M1 differentiation by suppressing inflammatory gene expression [60]. Finally, Jiang et al. [63] identified miR-9, miR-23a-3p, and miR-30d-5p as drivers of M1 pro-inflammatory macrophage polarization, while miR-124, miR-132, and miR-223 promoted M2 anti-inflammatory profiles [64]. All these studies illustrate that the ncRNA/macrophage axis is a critical determinant of host outcome in sepsis.

Sepsis-induced cardiac dysfunction (SICD) is a common and deadly complication of sepsis, contributing to poor clinical outcomes. Accumulating evidence points to a significant role for non-coding RNAs—particularly miRNAs and lncRNAs—in modulating inflammation, apoptosis, and mitochondrial dysfunction in the heart during sepsis. Manetti et al. identified several cardiac-specific miRNAs—miR-208a, miR-133a, and miR-499—that are elevated in both animal models and patients with SICD [59]. These miRNAs were associated with impaired calcium handling and mitochondrial damage. Maiese et al. expanded this view by analyzing miRNA signatures in sepsis-related organ dysfunction. They reported that reduced levels of miR-146a, miR-223, and let-7c were linked to worse cardiac, renal, and hepatic outcomes, suggesting that miRNA depletion may reflect the transition to an immunosuppressive state [61]. The role of lncRNAs in SICD has also been elucidated by Li et al., who focused on MIAT, HOTAIR, and TUG1. MIAT exacerbates myocardial inflammation via the miR-330-5p/NF-κB axis, while HOTAIR contributes to fibrosis through mitogen-activated protein kinase (MAPK) signaling. In contrast, TUG1 has a protective effect by enhancing SIRT1 activity and preserving mitochondrial integrity [65]. m^6^A RNA methylation has also been linked to cardiac dysfunction in sepsis. Wang et al. found that METTL3-mediated m^6^A methylation destabilizes calcium regulatory proteins and mitochondrial transcripts, aggravating cardiomyocyte apoptosis. Conversely, increasing FTO expression mitigated injury by reducing m^6^A levels and normalizing calcium flux [67]. Collectively, these findings underscore the central role of RNA-based regulation in sepsis-induced cardiac pathology and point to a promising future for RNA-targeted interventions in critical care cardiology. Despite these promising findings, the clinical interpretation of ncRNAs remains challenging. For instance, miRNAs such as miR-21, miR-125b, and miR-181 exhibit dual behavior—promoting either pro- or anti-inflammatory responses depending on timing, cell type, and disease phase—thereby complicating their use as consistent biomarkers. Similarly, lncRNAs like NEAT1 and MALAT1, although consistently upregulated in septic patients, may play either deleterious or compensatory roles depending on the inflammatory context. These inconsistencies highlight the need for functional studies and longitudinal profiling to clarify their mechanistic impact. The main aspects of nc-RNA regulation are summarized in Table 2.

## 9. Clinical Aspects: Patient Endotypes

One of the major obstacles in sepsis treatment is its extreme biological and clinical heterogeneity, which undermines the efficacy of uniform therapeutic strategies. To overcome this, the emerging concept of “endotyping” (the classification of patients into subgroups based on underlying molecular and immunological mechanisms) has gained consensus. Recent research suggests that epigenetic mechanisms, particularly gene expression and DNA methylation profiles, can be used to identify biologically meaningful endotypes in sepsis. Scicluna et al. [68] pioneered genomic endotyping in sepsis by analyzing whole blood transcriptomic data from over 700 patients and identifying four distinct modular acute response system endotypes (MARS1–4) using a 140-gene classifier. These molecular profiles were validated in multiple cohorts and correlated with differential mortality. MARS1, marked by suppression of genes related to innate and adaptive immunity and upregulation of metabolic genes, was associated with the highest mortality. In contrast, MARS3 displayed heightened adaptive immune signatures and was linked to better outcomes. The authors further derived an 8-gene signature for bedside endotype identification, demonstrating feasibility for clinical implementation. Davenport et al. [69] identified two sepsis response signatures (SRS1 and SRS2) in patients with pneumonia-related sepsis using unsupervised clustering of whole blood transcriptomes. SRS1, associated with increased early mortality, was defined by suppression of HLA class II genes and T-cell activation markers, suggesting a state of immunosuppression. Interestingly, this response was further modulated by context-specific expression quantitative trait loci (eQTL), highlighting the role of host genetic and epigenetic interactions in modulating sepsis prognosis. Similarly, Sweeney et al. [70] consolidated transcriptomic data from 14 independent sepsis cohorts to define three reproducible endotypes: inflammopathic, adaptive, and coagulopathic. The Inflammopathic endotype showed upregulation of innate immune pathways and higher mortality, whereas the adaptive endotype was characterized by interferon signaling and lower mortality. The coagulopathic endotype exhibited enrichment for genes related to clotting and poor prognosis in elderly patients. While transcriptomics provides a dynamic snapshot of the immune response, epigenetic modifications such as DNA methylation offer insights into more stable regulatory changes. Binnie et al. [71] conducted an epigenome-wide association study (EWAS) in sepsis and identified 668 differentially methylated regions (DMRs), many located in genes involved in antigen presentation (e.g., HLA-DQB1), interferon signaling, and complement activation. These DMRs also correlated with disease severity and clinical outcomes, reinforcing the idea that epigenetic programming contributes to sepsis heterogeneity. López-Cruz et al. [72] demonstrated that methylation changes, particularly in immune regulatory genes like serine protease inhibitor, clade A, member 1 (*SERPINA1*), and myeloperoxidase (*MPO*), can distinguish septic from non-septic patients with similar infections. They found that hypermethylation affected histone modifiers and interferon response elements, disrupting immune homeostasis. Network analysis revealed tight interactions among epigenetically altered genes, especially in the MHC locus on chromosome 6. Validation against transcriptomic datasets confirmed that a large proportion of DMR-associated genes also exhibited differential expression, suggesting functional impact. The role of epigenetics in immune cell differentiation during sepsis was further clarified by Kwok et al. [73]. Using single-cell transcriptomics and ATAC-seq (assay for transposase-accessible chromatin using sequencing), the study revealed how emergency granulopoiesis reprograms hematopoietic progenitors into immunosuppressive neutrophils in SRS1 patients. This process is driven by the transcription factors STAT3 and CCAAT/enhancer-binding protein beta (CEBPB). These neutrophils inhibited T-cell proliferation and contributed to immune paralysis. The epigenetic memory established in hematopoietic stem cells was persistent and linked to poor outcomes, suggesting that targeting this reprogramming may be a therapeutic option. Temporal profiling of gene expression provides additional granularity in endotype classification. Severino et al. [74] studied septic patients with community-acquired pneumonia and showed that non-survivors exhibited a decline in immune gene expression and mitochondrial function over 7 days, in contrast to sustained responses in survivors. Mitochondrial dysfunction, particularly involving electron transport chain components, emerged as a potential biomarker of poor prognosis. Burnham et al. [75] validated the concept of SRS dynamics by demonstrating that nearly half of the patients switched from the high-risk SRS1 to the lower-risk SRS2 profile within five days. Those who persisted in SRS1 had higher mortality, emphasizing the need for serial transcriptomic assessments to monitor disease progression and therapeutic response. However, while transcriptomic-based patient stratifications, as shown by Burnham et al. and Severino et al., propose that immune endotypes may inform prognosis and guide therapy, De Backer et al. [2] emphasize that our understanding of epigenetic drivers remains limited and temporally dynamic, complicating biomarker validation.

Recent studies have integrated transcriptomic, proteomic, and metabolomic data to refine endotyping. Balch et al. [76] used 33-gene signatures to define adaptive, inflammopathic, and coagulopathic endotypes in both septic and non-septic ICU patients. Septic patients with inflammopathic and coagulopathic profiles had significantly higher secondary infection rates, while adaptive endotype patients had better outcomes. The study emphasized the dynamic nature of endotypes, recommending repeated profiling during ICU stay. Bodinier et al. [77] proposed a transcriptomic score (TScore) based on eight genes to stratify ICU patients by infection risk. High TScore (≥3) patients had more ICU-acquired infections, longer stays, and higher mortality. This group also showed reduced monocytic HLA-DR and elevated IL-10, reflecting immunosuppression. The score was validated in multiple cohorts and proposed as a tool for identifying candidates for immunotherapy. Baghela et al. [78] identified five endotypes at first clinical presentation in the emergency room: Neutrophilic-Suppressive (NPS), Inflammatory (INF), Innate Host Defense (IHD), Interferon (IFN), and Adaptive (ADA). These endotypes were retained in ICU patients and correlated with severity metrics such as SOFA scores and hospital stay duration. The NPS and INF endotypes were linked to neutrophilia, immunosuppression, and high mortality. Notably, STAT3 emerged as a central regulator, particularly in the NPS endotype. The early stratification of patients opens opportunities for personalized interventions before progression to critical illness. Beyond DNA methylation, RNA modifications like m^6^A are increasingly recognized as epigenetic regulators. Zhang et al. [79] analyzed expression profiles of 21 m^6^A-related genes in sepsis and classified patients into two endotypes: Cluster A, with high m^6^A regulator expression and strong immune activation, and Cluster B, with low expression and immune exhaustion. Cluster A has been associated with better survival, while Cluster B was linked to regulatory T cells and M2 macrophages, indicating immunosuppression. These findings suggest that m^6^A modification profiles can serve as both diagnostic markers and therapeutic targets in sepsis. The classification of sepsis patients into molecular endotypes based on epigenetic biomarkers has evolved from a theoretical framework into a clinically actionable strategy. Across multiple studies, endotypes have been consistently linked to severity outcomes and responses to therapy. These endotypes are shaped by a combination of transcriptomic patterns, DNA and RNA methylation, chromatin accessibility, and genetic variants that regulate immune gene expression. These insights have deep implications for precision medicine in sepsis. First, endotypes could guide patient selection in clinical trials, reducing the failure of “one-size-fits-all” interventions. Second, they enable targeted therapies—immune stimulants for immunosuppressed patients, anti-inflammatories for hyperinflammatory ones, or *STAT3* inhibitors for those with epigenetic neutrophil reprogramming. Finally, rapid, bedside-compatible platforms (e.g., qRT-PCR for transcriptomic signatures or methylation panels) will be essential for translating these findings into practice.

## 10. Epigenetic Modifications as Biomarkers

The clinical utility of epigenetic biomarkers in sepsis has gained increasing attention due to their potential to stratify patients, predict outcomes, and guide therapy. DNA methylation, particularly at CpG islands within promoter regions, represents the most studied epigenetic regulatory mechanism. Several studies provide compelling evidence for its role in modulating immune responses during sepsis. Beltrán-García et al. showed that leukocyte DNA methylation profiles are deeply altered in patients with sepsis and septic shock, identifying over 1600 differentially methylated regions (DMRs) in septic shock patients compared to critically ill controls. Methylation changes in promoters of immune-related genes such as *IL-10*, *IL-1B*, and *TREM1* correlated with immunosuppression and organ dysfunction, offering a promising epigenetic signature for severity assessment and prognosis [31]. Further supporting the diagnostic potential of methylation patterns, Falcão-Holanda et al. reported 668 DMRs between septic and non-septic patients, including genes already implicated in sepsis pathogenesis. The study also noted specific methylation alterations in the calcitonin-related polypeptide alpha (*CALCA*) gene promoter in preterm neonates, correlating with infection type, indicating potential roles for such epigenetic profiles in infection-type stratification [4]. Moreover, Ma et al. reported that hypermethylation of the *AQP5* promoter in neutrophils from sepsis patients negatively associates with AQP5 expression and protective immune responses, suggesting AQP5 methylation as a candidate biomarker and therapeutic target [28]. In septic patients, specific histone marks dynamically regulate the expression of pro- and anti-inflammatory genes. Increased H3K4me3 and H3K9ac at immune response genes, and enrichment of H3K27me3 at antigen presentation genes, were observed in chromatin immunoprecipitation sequencing (ChIP-seq) analyses [4]. These chromatin signatures reflect immune activation or repression states, suggesting histone modifications could serve as biomarkers of immune function during different sepsis stages. Davis et al. showed that sepsis leads to reduced H3K4me3 levels at NF-κB–binding sites and decreased expression of MLL1, a histone methyltransferase, in both bone marrow and wound macrophages. This epigenetic suppression persisted long after clinical recovery and was associated with impaired inflammation and wound healing [41]. These findings underscore the utility of histone modification profiles in evaluating long-term immunological sequelae in sepsis survivors, which could inform post-discharge risk monitoring and interventions. Non-coding RNAs may also exert a regulatory effect in sepsis and hold substantial promise as clinical biomarkers. According to Falcão-Holanda et al., transcriptomic profiling of septic patients revealed significant alterations in both lncRNA and miRNA expression, particularly in neutrophils and circulating leukocytes [4]. These molecules regulate several pathways involved in NF-κB signaling and cytokine production and may function as upstream controllers of gene expression changes during sepsis. Specific miRNAs, such as miR-146a and miR-155, are already known to modulate sepsis-relevant pathways, and their circulating levels may reflect disease stage and prognosis. A number of miRNAs have emerged as potential biomarkers. Circulating miR-150 inversely correlates with SOFA scores, while miR-25 has shown superior diagnostic accuracy compared to CRP and PCT in some cohorts [8]. However, further studies are necessary to validate their clinical utility. Cell-free DNA (cfDNA), particularly its methylation profile, is another emerging epigenetic biomarker. Ma et al. reported that cfDNA levels in septic patients correlate with disease severity and mortality risk. Advances in single-cell methylome deconvolution now enable tracing cfDNA to specific immune cell lineages, revealing elevated cfDNA originating predominantly from granulocytes in sepsis. This suggests active neutrophil engagement and lysis, providing a non-invasive method to assess immune response patterns in sepsis [28]. Epigenetic reprogramming is also central to the phenomena of endotoxin tolerance and trained immunity. Venet and Monneret described how loss of activating histone marks (e.g., H3K4me) and gain of repressive marks (e.g., H3K9me2) silence inflammatory genes during the tolerogenic phase of sepsis, contributing to immune suppression [26]. Conversely, trained immunity involves epigenetic priming (e.g., H3K4me3 enrichment) of pro-inflammatory gene promoters, enhancing pathogen resistance. The presence or absence of these chromatin marks may thus serve as temporal biomarkers indicating immune dynamics and risk of secondary infections. Despite these advances, challenges remain in translating epigenetic biomarkers into routine clinical practice. Technical variability in sample processing, the need for high-throughput and cost-effective detection platforms, and the temporal instability of some epigenetic marks are critical obstacles. Moreover, as highlighted in the Surviving Sepsis Campaign Research Priorities, our understanding of how epigenetic landscapes evolve over time in sepsis is still limited, necessitating longitudinal studies to validate candidate biomarkers [2]. Epigenetic biomarkers, ranging from DNA methylation and histone modifications to ncRNA expression and cfDNA methylation profiles, offer a multidimensional toolkit for improving sepsis diagnosis, prognosis, and treatment personalization. The dynamic, cell-type-specific, and reversible nature of these modifications makes them particularly attractive for real-time monitoring and therapeutic targeting. As high-resolution and single-cell technologies continue to advance, the integration of epigenetic biomarkers into precision medicine frameworks for sepsis appears both feasible and imminent.

## 11. Influence on Therapy Response

Despite advancements in supportive care and antibiotics, the efficacy of immunomodulatory or adjunctive therapies in sepsis has remained inconsistent across clinical trials. This inconsistency reflects, in part, the huge biological heterogeneity among septic patients, which has increased the need for molecular biomarkers capable of stratifying patients according to their likely response to treatment. In this context, epigenetic modifications have emerged as promising predictors of therapeutic responsiveness. Ma et al. investigated methylation in neutrophils and found that the *AQP5* gene, a water channel involved in neutrophil migration and immune defense, was hypermethylated in sepsis patients. The degree of *AQP5* promoter methylation inversely correlated with gene expression and clinical outcomes. Functionally, AQP5 helps regulate the balance between neutrophil activation and inflammation resolution. Patients with hypermethylated *AQP5* may thus form a distinct subgroup with impaired neutrophil responses. This subgroup could show reduced efficacy to neutrophil-dependent therapies but might benefit from granulocyte colony-stimulating factor (G-CSF) administration. Furthermore, DNA methylation patterns could be used to predict responsiveness to demethylating agents such as decitabine, which have shown promise in preclinical models for reversing immune cell exhaustion [28]. Histone deacetylase inhibitors (HDACi) such as trichostatin A (TSA), valproic acid, and suberoylanilide hydroxamic acid (SAHA) have shown therapeutic potential in animal models of sepsis. These agents promote acetylation of histones, leading to increased expression of genes involved in immune tolerance, cellular survival, and mitochondrial function. Falcão-Holanda et al. summarized multiple studies showing that HDACi administration in septic rodents improved survival, reduced cytokine storms, and restored mitochondrial homeostasis [4]. Importantly, the baseline histone acetylation status or HDAC expression levels in patient immune cells could serve as predictive biomarkers to determine who may benefit most from HDACi therapy. Among the most clinically validated applications of epigenetics-informed therapy in sepsis is the use of transcriptomic endotyping to guide corticosteroid treatment. In a secondary analysis of the VANISH trial, Antcliffe et al. identified two major transcriptomic endotypes in sepsis: SRS1 and SRS2 [80]. SRS1 was characterized by suppression of adaptive immunity, decreased expression of HLA-DR and T-cell signaling genes, and features of endotoxin tolerance. In contrast, SRS2 exhibited preserved immune competence and inflammatory signaling. Interestingly, the interaction between SRS subtype and corticosteroid treatment was striking: SRS2 patients treated with hydrocortisone experienced significantly increased 28-day mortality compared to the placebo (harf ratio 7.9, 95% confidence intervals 1.6–39.9), whereas SRS1 patients did not show this harmful effect. This suggests that immunocompetent patients (SRS2) may be harmed by additional immunosuppression, while immunoparalyzed patients (SRS1) could potentially benefit or at least avoid harm. These findings highlight transcriptomic profiling as a potential real-time clinical tool for therapy guidance. Notably, many genes in the SRS signatures are epigenetically regulated, including those controlled by DNA methylation or chromatin accessibility. This suggests epigenomic assays could act as faster and more cost-effective alternatives to transcriptomic classifiers. Integration of methylation arrays, ChIP-seq, or ATAC-seq into point-of-care platforms could further enhance the feasibility of endotype-guided corticosteroid use. The relationship between epigenetics and cellular metabolism is bidirectional and tightly linked. Metabolites such as NAD⁺, acetyl-CoA, and alpha-ketoglutarate serve as cofactors or inhibitors of epigenetic enzymes, and their availability influences histone acetylation, DNA methylation, and RNA modifications. Conversely, epigenetic changes modulate the transcription of metabolic enzymes and transporters. Puskarich et al. employed a pharmacometabolomic approach to identify metabolic endotypes responsive to L-carnitine therapy in septic shock [81]. Their trial revealed that septic patients with baseline serum acetylcarnitine (C2) levels ≥35 μM had a 14% absolute reduction in 90-day mortality when treated with 18 g of intravenous L-carnitine, compared to those with lower levels. These patients likely had mitochondrial dysfunction and impaired beta-oxidation, making them more amenable to carnitine supplementation. Importantly, this stratification could not be achieved with traditional clinical markers such as SOFA scores or lactate levels. These findings raise the possibility that acetylcarnitine levels—and the underlying mitochondrial epigenetic profile—could be used to guide metabolic resuscitation strategies. Since epigenetic enzymes such as sirtuins and histone acetyltransferases rely on mitochondrial-derived cofactors, integration of metabolomic and epigenetic profiling may provide a more holistic view of cellular fitness and therapy responsiveness in sepsis. Among the most recent and promising developments in epigenetic regulation is the study of RNA modifications, particularly m^6^A. This abundant mRNA modification affects transcript stability, splicing, and translation. Qian et al. analyzed transcriptomic data from over 800 sepsis patients and identified two distinct m^6^A methylation clusters based on the expression of “writers” (e.g., METTL3, METTL14), “erasers” (e.g., FTO, ALKBH5), and “readers” (e.g., YTHDF1, IGF2BP2) [82]. Cluster A, characterized by higher expression of methyltransferases, showed enrichment of regulatory immune cell subsets (e.g., Tregs, resting dendritic cells), while Cluster B, with elevated expression of reader proteins, exhibited an inflammatory cell profile dominated by M1 macrophages and neutrophils. These clusters also differed in activation of immune pathways such as Toll-like receptor signaling, NF-κB activation, and cytokine–cytokine receptor interactions. The authors proposed that Cluster B patients may experience hyperinflammation and cytokine storms due to unregulated mRNA translation of pro-inflammatory cytokines driven by YTHDF1. In contrast, Cluster A may be more susceptible to immunosuppression and impaired microbial clearance.

This m^6^A-based stratification opens new options for RNA-targeted therapies in sepsis. For instance, inhibitors of METTL3 or YTHDF1 may turn down overactive inflammation in Cluster B patients, while demethylase activators could restore immune competence in Cluster A. Moreover, RNA methylation status could be used as a predictive biomarker for immunotherapies such as checkpoint inhibitors or adoptive T-cell therapies, especially in patients with post-septic immunoparalysis. Table 3 summarizes these clinical aspects.

## 12. Epigenetic-Targeted Therapies

Targeting epigenetic mechanisms in sepsis has emerged as a promising therapeutic strategy to modulate the dysregulated immune response and organ dysfunction that characterize the syndrome. Histone deacetylase inhibitors (HDACi) such as trichostatin A (TSA), valproic acid, and suberoylanilide hydroxamic acid (SAHA) have shown therapeutic potential in animal models of sepsis. These agents promote acetylation of histones, leading to increased expression of genes involved in immune tolerance, cellular survival, and mitochondrial function. Falcão-Holanda et al. summarized multiple studies showing that HDACi administration in septic rodents improved survival, reduced cytokine storms, and restored mitochondrial homeostasis [4]. Importantly, the baseline histone acetylation status or HDAC expression levels in patient immune cells could serve as predictive biomarkers to determine who may benefit most from HDACi therapy.

Zhang et al. [83] demonstrated that administration of pan-HDAC inhibitors, such as trichostatin A (TSA) and sodium butyrate, significantly attenuated acute lung injury (ALI) following cecal ligation and puncture (CLP) in mice by reducing neutrophil infiltration, pro-inflammatory cytokines (TNF-α, IL-6), and histological damage. Their study notably showed improved 7-day survival, linking histone acetylation to decreased systemic inflammation and preservation of lung function. Extending this, Takebe et al. [84] found that HDACi, including valproic acid (VPA) and the novel inhibitor CG200745, mitigated apoptosis in lungs and spleen during sepsis, highlighting organ-protective effects without systemic immunosuppression. This organ-specific cytoprotection underscores the therapeutic potential of HDAC inhibition to preserve immune organ integrity and function during sepsis. Refining this approach, Zhang et al. [85] focused on selective HDAC6 inhibition using tubastatin A in a CLP-induced sepsis model. They reported significant reductions in lung injury, vascular permeability, and pro-inflammatory cytokines without compromising bacterial clearance, suggesting that isoform-selective HDACi may maximize anti-inflammatory benefits while minimizing immunosuppressive risks. Similarly, Williams et al. [86] and von Knethen et al. [87] reviewed the broad potential of both pan and selective HDACi in trauma and sepsis, emphasizing the importance of timing due to the biphasic immune response in sepsis—hyperinflammation followed by immunosuppression. They stressed that indiscriminate HDAC inhibition risks impairing host defense during late sepsis and advocated for patient stratification guided by biomarkers to optimize therapeutic windows.

Beyond pan and HDAC6 inhibitors, targeting class III HDACs (sirtuins) represents a novel avenue. Zhao et al. [88] demonstrated that nicotinamide, a pan-sirtuin inhibitor, improved survival and hemodynamic stability in lethal sepsis models by modulating inflammatory cytokines and preserving mitochondrial function. This intervention targets metabolic and inflammatory pathways pivotal to sepsis pathophysiology, suggesting sirtuin modulation as a promising adjunctive therapy. Parallel to histone acetylation, DNA methylation plays a crucial role in sepsis immunoregulation. Cao et al. [89] provided compelling evidence that DNA methyltransferase inhibition using 5-aza-2′-deoxycytidine (decitabine) improved survival in CLP-induced severe sepsis by attenuating NF-κB pathway activation and reducing pro-inflammatory cytokine release. The treatment reduced expression of DNMT1, DNMT3a, and DNMT3b, partially reversing aberrant DNA methylation and restoring more balanced immune responses. These findings suggest that targeting epigenetic dysregulation via DNMT inhibition may help prevent immune overactivation and mitigate organ injury in sepsis. Complementary to this, Beltrán-García et al. [25] reviewed epigenetic biomarkers, including DNA methylation patterns, as tools to guide personalized therapies in sepsis. They highlighted that epigenetic silencing of immune genes such as HLA-DR via hypermethylation contributes to immunoparalysis, and reversing these marks with DNMTi could restore immune competence, particularly in immunosuppressed sepsis endotypes. The review also emphasized the potential of epigenetic profiling to predict responses to conventional therapies like corticosteroids, underscoring the utility of epigenetic-guided precision medicine. RNA-based epigenetic therapeutics have also gained momentum. Dragomir et al. [90] investigated the inhibition of microRNA-93-5p (miR-93-5p), which is upregulated in sepsis and suppresses immune activation genes. Their anti-miR-93-5p therapy in murine and non-human primate sepsis models enhanced T-cell function, cytokine production, bacterial clearance, and survival without exacerbating inflammation. This study pioneers the use of RNA-targeted epigenetic modulation to reverse sepsis-induced immune dysfunction and suggests RNA interference (RNAi) or antisense oligonucleotides as viable therapeutic platforms. These approaches complement existing HDAC and DNMT inhibitors by targeting non-coding RNAs that regulate gene networks controlling immune responses. Several preclinical studies highlight the therapeutic potential of targeting microRNAs to modulate immune responses in sepsis. Importantly, RNA-based therapies offer the advantage of high specificity, reversible modulation, and potential for systemic or tissue-targeted delivery, which may reduce off-target effects and immune suppression risks typical of broad epigenetic drugs.

However, clinical translation of epigenetic therapies in sepsis faces substantial challenges. The complexity of sepsis immunopathology, with rapid and heterogeneous shifts from hyperinflammation to immunoparalysis, demands precise timing and patient stratification. HDAC inhibitors can impair host defense if administered during late immunosuppression, while DNA methylation inhibitors require optimization to avoid global hypomethylation and genomic instability. Additionally, pharmacokinetics of epigenetic drugs in critically ill patients remain poorly defined, and the development of isoform-selective and tissue-specific agents is ongoing to improve safety and efficacy.

Integration of epigenetic biomarkers, such as DNA methylation signatures and histone modification profiles, holds promise for personalized approaches. This could enable real-time identification of immune phases and guide selection of epigenetic modulators tailored to individual patients’ immune status. Moreover, combining epigenetic drugs with conventional therapies, such as antibiotics, corticosteroids, or immune checkpoint inhibitors, may enhance therapeutic synergy and mitigate resistance mechanisms.

In conclusion, therapeutic targeting of epigenetic regulators in sepsis—including histone acetylation via HDAC inhibition, DNA methylation modulation by DNMT inhibitors, and RNA-based epigenetic therapies—represents a multifaceted approach to restore immune homeostasis, limit organ damage, and improve survival. While preclinical data are compelling and span multiple epigenetic layers, clinical translation requires further development of selective compounds, biomarker-driven patient stratification, and optimized dosing strategies. Continued research integrating epigenomic profiling and RNA therapeutics is likely to expand the therapeutic arsenal for this complex and heterogeneous syndrome.

## 13. Conclusions

Collectively, the integration of DNA methylation profiles, histone modification landscapes, transcriptomic and metabolic endotypes, and RNA methylation patterns presents a powerful toolkit for precision medicine in sepsis. These biomarkers can stratify patients into biologically distinct subgroups that respond differently to corticosteroids, metabolic therapies, immunomodulators, or epigenetic drugs. However, despite promising advances, several unresolved issues remain. Epigenetic data across studies often show conflicting patterns, likely reflecting the influence of timing, cell type, and patient heterogeneity. The dual roles of certain non-coding RNAs, the context-dependent function of histone modifications, and the ambiguity in interpreting DNA methylation changes—as either adaptive or pathogenic—pose major challenges. Furthermore, experimental therapies targeting epigenetic pathways have shown variable efficacy in preclinical models and are not yet ready for clinical translation. To implement such strategies in clinical practice, several challenges must be addressed. First, rapid and cost-effective epigenetic assays suitable for bedside use are needed. Second, prospective trials incorporating epigenetic stratification must validate predictive biomarkers in diverse populations. Particularly important is the validation of candidate epigenetic biomarkers in larger, multicenter cohorts with longitudinal sampling to assess their reproducibility and prognostic value over time. Third, bioinformatics tools for integrating multi-omic data must be standardized and user-friendly. Lastly, ethical considerations around genomic and epigenomic testing in acutely ill patients must be carefully navigated. Nonetheless, the direction of current research indicates that the future of sepsis care lies in a shift from syndrome-based to mechanism-based treatment paradigms. Epigenetics will likely be essential in this transition, enabling more targeted, effective, and personalized therapies for one of medicine’s most formidable challenges.

## Figures and Tables

**Table 1:** Major Histone Modifications and Their Pathophysiological Effects in Sepsis.

Histone Modification	Mechanism	Target Genes/Pathways	Pathophysiological Effect
H3K27me3	Catalyzed by EZH2; repressive mark	*Sox9*, *IL-12* promoter	Suppresses gene expression; associated with immune cell dysfunction, AKI and reduced IL-12 in dendritic cells [42,50]
H3K4me3	Activating mark at gene promoters	NF-κB target genes	Enhances transcription of inflammatory genes; its loss linked to long-term immune suppression post-sepsis [4,41]
H3K9ac/H4ac	Acetylation by HATs increases chromatin accessibility	*Foxp3*, cytokine promoters	Promotes transcription; H3K9ac linked to Treg polarization and immunosuppression [4]
H3K18la	Addition of lactyl group from lactate via p300	*EGR1*, *RhoA*	Promotes transcription of genes involved in repair and inflammation; associated with ALI, AKI, and immunosuppression [46,47,48,51]
Citrullination	Converts arginine to citrulline	NET-related genes	Enables NET formation; contributes to hyperinflammation and tissue damage [6,38]
Deacetylation	Removes acetyl groups; represses gene transcription	*TNF*, *IL-1β*	Promotes immunosuppression; HDAC1/2 modulates TLR responses and contributes to endotoxin tolerance [6,39]

Legend: EZH2: enhancer of zeste 2 polycomb repressive complex 2 subunit, *Sox9*: SRY-Box Transcription Factor 9, AKI: acute kidney injury, NF-κB: nuclear factor kappa B, HATs: histone acetyltransferases, *Foxp3*: forkhead box P3, *EGR1*: early growth response protein 1, ALI: acute lung injury, NET: neutrophil extracellular trap, *TNF*: tumor necrosis factor, HDAC1/2: histone deacetylase 1/2, TLR: toll-like receptor.

**Table 2:** Non-Coding RNAs in Sepsis: Molecular Functions and Pathophysiological Roles.

ncRNA Type/Example	Molecular Function/Target	Immune/Cellular Effects	Pathophysiological/Clinical Relevance
miR-146a	Targets *IRAK1*, *TRAF6* in TLR/NF-κB pathway	Suppresses inflammatory signaling	Protects against cytokine storm and cardiac dysfunction [8,37,60,61]
miR-155	Inhibits *SOCS1*	Enhances NF-κB activation, promotes M1 macrophage polarization	Amplifies early inflammatory response [58]
miR-223	Regulates *NLRP3*, *RhoB*	Promotes M2 macrophage phenotype; modulates neutrophil activation	Linked to improved survival in murine sepsis models [30,61,64]
miR-125b	Represses *Bcl6*, activates JNK pathway	Drives M1 polarization	Contributes to early inflammation [8,37]
miR-150	General immunoregulatory role; diagnostic value	Reduced levels associated with worse outcome	Circulating biomarker correlating with SOFA score [8,58]
lncRNA NEAT1	Sponges miR-125a-5p, derepresses *TRAF6*	Promotes M1 polarization and NF-κB activation	Pro-inflammatory role; marker of immune dysregulation [37]
lncRNA MALAT1	Sponges miR-150-5p	Upregulates *ICAM-1*, promotes endothelial activation	Aggravates sepsis-induced acute lung injury (ALI) [58]
lncRNA TUG1	Activates *SIRT1* via miR-9-5p axis	Promotes M2 polarization, stabilizes mitochondrial function	Protective in sepsis; enhances survival and tissue repair [65]
circRNA circPPM1F	Sponges miRNAs that inhibit NF-κB	Promotes M1 macrophage polarization	Exacerbates cytokine storm [60]
circRNA circ_0038644	Inhibits IL-6 production	Supports anti-inflammatory profile	Protective effect during hyperinflammation [60]

Legend: IRAK1: Interleukin-1 Receptor-Associated Kinase 1, TRAF6: Tumor Necrosis Factor Receptor-Associated Factor 6, TLR/NF-κB: toll-like receptor/nuclear factor kappa B, SOCS1: suppressor of cytokine signaling 1, NLRP3: NOD-, LRR- and pyrin domain-containing protein 3, RhoB: Ras Homolog Family Member B, Bcl6: B-cell lymphoma 6, JNK: c-Jun N-terminal kinases, ICAM-1: intercellular adhesion molecule 1, SIRT1: sirtuin 1, miRNAs: micro-RNAs.

**Table 3:** Epigenetic Endotypes and Predictive Biomarkers of Therapy Response in Sepsis.

Biomarker/Endotype	Molecular Characteristics	Associated Therapy Response
SRS1 (Sepsis Response Signature 1)	Suppression of HLA-DR and T-cell signaling; epigenetic repression of immune genes	No benefit or potential benefit from corticosteroids; potential target for immune-stimulatory therapies [69,76,80]
SRS2 (Sepsis Response Signature 2)	Preserved immune function; high expression of adaptive immune genes	Corticosteroids associated with significantly increased 28-day mortality [69,80]
AQP5 DNAMethylation	Hypermethylation of *AQP5* promoter in neutrophils reduces gene expression and cell migration	Predicts poor outcome and reduced response to neutrophil-targeting therapies (e.g., G-CSF) [28]
Histone Acetylation/HDAC Activity	Altered levels of histone acetylation (e.g., reduced H3K9ac, increased HDAC activity) suppress immune gene transcription	Response to histone deacetylase inhibitors (HDACi) in preclinical models [6,80]
Acetyl carnitine Levels ≥ 35 µM	Reflects mitochondrial dysfunction and metabolic distress	Identifies responders to L-carnitine therapy; associated with reduced 90-day mortality in treated patients [81]
m^6^A Cluster A	High expression of *METTL3*, enrichment in Tregs and resting dendritic cells; immune-suppressive signature	May benefit from demethylase activators (e.g., FTO, ALKBH5) to restore immune competence [82]
m^6^A Cluster B	High *YTHDF1*, increased M1 macrophages and neutrophil activation; hyperinflammatory profile	Potential target for *METTL3* or *YTHDF1* inhibition to dampen cytokine storm [82]

Legend: HLA-DR: human leukocyte antigen DR, AQP5: aquaporin 5, G-CSF: granulocyte colony stimulating factor, HDAC: histone deacetylase, METTL3: N6-adenosine-methyltransferase 70 kDa subunit, FTO: fat mass and obesity-associated gene, ALKBH5: AlkB Homolog 5.

## Data Availability

Not applicable.

## Abbreviations

The following abbreviations are used in this manuscript:

DNMT	DNA methyltransferases
CpG	cytosine residues adjacent to guanines
5mC	5-methylcytosine
PAD	peptidyl arginine deaminase
HPTM	histone post-translational modifications
ncRNA	non-coding RNA
miRNA	MicroRNAs
lncRNA	long non-coding RNA
circRNA	circular RNA
TNFα	tumor necrosis factor alfa
IL	interleukine
CARS	compensatory anti-inflammatory response syndrome
PICS	persistent inflammation, immunosuppression, and catabolism syndrome
Tregs	regulatory T cells
MDSCs	myeloid-derived suppressor cells
Foxp3+	forkhead box P3
TGF-β	transforming grow factor-β
TIM-3	T-cell immunoglobulin and mucin-domain containing-3
PD-1	programmed cell death protein 1
TIGIT	T cell immunoreceptor with Ig and ITIM domains
CTLA-4	cytotoxic T-lymphocyte antigen 4
MHC	major histocompatibility complex
JAK3	janus kinase 3
STAT5	signal transducer and activator of transcription 5
LPS	lipopolysaccharide
GPR84	G protein-coupled receptor 84
NF-κB	nuclear factor kappa-light-chain-enhancer of activated B cells
DAMPs	damage-associated molecular patterns
PAMPS	pathogen-associated molecular patterns
NEAT1	Nuclear Paraspeckle Assembly Transcript
m^6^A	N6-methyladenosine
METTL3	methyltransferase-like 3
FTO	fat mass and obesity-associated protein
YTHDF1	YTH N6-Methyladenosine RNA Binding Protein 1
NETs	neutrophil extracellular traps
AKI	acute kidney injury
ALI	acute lung injury
SICD	sepsis-induced cardiac dysfunction
AQP5	aquaporin-5
MERS	Middle East respiratory syndrome
CoV	Coronavirus
TREM1	triggering receptor expressed on myeloid cells 1
TNFAIP8	TNFα-Induced Protein 8
SIRS	systemic inflammatory response syndrome
SOFA	sequential organ failure assessment
TLR4	toll-like receptor 4
PADs	peptidylarginine deaminases
HDAC1	histone deacetylase 1
JMJD3	jumonji domain-containing protein 3
ACLY	ATP citrate lyase
MLL1	mixed-lineage leukemia 1
GATA3	guanine–adenine–thymine–adenine binding protein 3
LLO	listeriolysin O
NUE	nuclear ubiquitin E3 ligase
OspF	outer surface protein F
IpaH9.8	invasion plasmid antigen H 9.8
Kla	lysine lactylation
APACHE	acute physiology and chronic health evaluation
ICU	intensive care unit
CLP	cecal ligation and puncture
EZH2	enhancer of zeste 2 polycomb repressive complex 2 subunit
Sox9	SRY-box transcription factor 9
EGR1	early growth response protein 1
HPSE	heparanase
ARDS	acute distress respiratory syndrome
SIRT	sirtuin
NAD	nicotinamide adenine dinucleotide
IRAK1	IL-1 receptor-associated kinase 1
TRAF6	TNF receptor-associated factor 6
NLRP3	NOD-, LRR-, and Pyrin domain-containing protein 3
SOCS1	suppressor of cytokine signaling 1
Bcl6	B-cell lymphoma 6
JNK	c-Jun N-terminal kinase
CRP	C-reactive protein
NEAT1	nuclear-enriched abundant transcript 1
RhoA	Ras homolog gene family A
ROCK	Rho-associated coiled-coil-containing protein kinase
MALAT1	metastasis-associated lung adenocarcinoma transcript 1
ICAM-1	intercellular adhesion molecule 1
TUG1	taurine upregulated gene 1
MIAT	myocardial infarction-associated transcript
HOTAIR	HOX transcript antisense intergenic RNA
circ-RSF1	circular remodeling and spacing factor 1
PPM1F	protein phosphatase, Mg2+/Mn2+ dependent 1F
EPC	endothelial progenitor cells
ALKBH5	AlkB homolog 5, RNA demethylase
MAPK	mitogen-activated protein kinase
MARS	modular acute response system
SRS1	sepsis response signatures
eQTL	expression quantitative trait loci
EWAS	epigenome-wide association study
DMRs	differentially methylated regions
SERPINA1	serine protease inhibitor, clade A, member 1
MPO	myeloperoxidase
ATAC-seq	assay for transposase-accessible chromatin using sequencing
CEBPB	CCAAT/enhancer-binding protein beta
NPS	Neutrophilic-Suppressive
INF	Inflammatory
IHD	Innate Host Defense
IFN	Interferon
ADA	Adaptive
CALCA	calcitonin-related polypeptide alpha
ChIP-seq	chromatin immunoprecipitation sequencing
PCT	procalcitonin
cfDNA	cell-free DNA
G-CSF	granulocyte colony-stimulating factor
HDACi	histone deacetylase inhibitors
SAHA	suberoylanilide hydroxamic acid
